# Maternal Vitamin D Status and Infant Outcomes in Rural Vietnam: A Prospective Cohort Study

**DOI:** 10.1371/journal.pone.0099005

**Published:** 2014-06-26

**Authors:** Sarah Hanieh, Tran T. Ha, Julie A. Simpson, Tran T. Thuy, Nguyen C. Khuong, Dang D. Thoang, Thach D. Tran, Tran Tuan, Jane Fisher, Beverley-Ann Biggs

**Affiliations:** 1 Department of Medicine, Melbourne Academic Centre, University of Melbourne at the Doherty Institute, Parkville, Victoria, Australia; 2 Research and Training Centre for Community Development (RTCCD), Hanoi, Vietnam; 3 Centre for Epidemiology and Biostatistics, Melbourne School of Population and Global Health, University of Melbourne, Parkville, Victoria, Australia; 4 Provincial Centre of Preventive Medicine, Hanam, Hanam Province, Vietnam; 5 The Jean Hailes Research Unit, School of Public Health and Preventive Medicine, Monash University, Clayton, Victoria, Australia; 6 The Victorian Infectious Diseases Service, Royal Melbourne Hospital, Parkville, Victoria, Australia; University of Queensland, Australia

## Abstract

**Objective:**

Vitamin D deficiency affects 1 billion people globally. It has an important role in bone homeostasis, brain development and modulation of the immune system and yet the impact of antenatal vitamin D deficiency on infant outcomes is poorly understood. We assessed the association of 25- hydroxyvitamin D levels (25-OHD) in late pregnancy and early infant growth and developmental outcomes in rural Vietnam.

**Design and Methods:**

A prospective cohort study of 960 women who had previously participated in a double-blind cluster randomized controlled trial of antenatal micronutrient supplementation in rural Vietnam was undertaken. Maternal 25-OHD concentration was measured at 32 weeks gestation, and infants were followed until 6 months of age. Main outcome measures were cognitive, motor, socio-emotional and language scores using the Bayley Scales of Infant Development, 3^rd^ edition, and infant length-for-age z scores at 6 months of age.

**Results:**

60% (582/960) of women had 25-OHD levels <75 nmol/L at 32 weeks gestation. Infants born to women with 25-OHD deficiency (<37.5 nmol/L) had reduced developmental language scores compared to those born to women who were vitamin D replete (≥75 nmol/L) (Mean Difference (MD) −3.48, 95% Confidence Interval (CI) −5.67 to −1.28). For every 25 nmol increase in 25-OHD concentration in late pregnancy, infant length-for-age z scores at 6 months of age decreased by 0.08 (95% CI −0.15 to −0.02).

**Conclusions:**

Low maternal 25- hydroxyvitamin D levels during late pregnancy are of concern in rural Vietnam, and are associated with reduced language developmental outcomes at 6 months of age. Our findings strengthen the evidence for giving vitamin D supplementation during pregnancy.

## Introduction

Vitamin D deficiency affects more than 1 billion people [1] and is now recognised as a major public health problem [2]. Important biological functions involving growth and developmental outcomes have been attributed to vitamin D, and deficiency during pregnancy may result in important health consequences for both mother and child [3], [4], [5].

Maternal vitamin D readily crosses the placenta, and maternal levels strongly correlate with infant vitamin D concentration at birth [6]. The major supply of vitamin D is through synthesis in the skin, following exposure to ultraviolet light. Dietary intake makes only a small contribution to vitamin D status [3], [7].

Although the current recommended dietary intake of vitamin D during pregnancy ranges from 600 to 2000 international units (IU) per day [8], a standardised definition of vitamin D deficiency during pregnancy remains controversial [4], [9], [10], [11]. A recent meta-analysis by Aghajafari et al.[12] categorised vitamin D insufficiency for pregnancy outcomes as serum 25-OHD concentration less than 75 nmol/L, and for vitamin D insufficiency for birth outcomes, as serum 25-OHD concentration less than 37.5 nmol/L [12]. Vitamin D supplementation for the prevention of pre-eclampsia and its complications during pregnancy was recently assessed by WHO and is currently not recommended [13], [14].

Adverse maternal and neonatal outcomes have previously been described in association with antenatal vitamin D deficiency, including increased risk of pre-eclampsia, gestational diabetes, caesarean section, as well as low birth weight and small for gestational age infants [15], [16], [17], [18], [19]. Vitamin D is also thought to effect bone formation and density and modulation of the immune system [5], [6], [20], [21], [22], [23]. More recently, maternal vitamin D deficiency has been associated with impaired infant language development in school-aged children [24], and has been suggested as a possible environmental risk factor for autism spectrum disorder, highlighting the important role of vitamin D in brain development, neuronal function and gene regulation [10], [25], [26].

Vitamin D insufficiency in Vietnam has previously been reported to be as high as 46% [27], [28], [29], however, there is limited data on the burden and consequences of vitamin D deficiency during pregnancy in this setting [17] and few studies have followed infants past the neonatal period. The role of vitamin D deficiency during pregnancy on longer term infant outcomes, particularly with regard to early childhood development is unclear.

We conducted a prospective cohort study, in a rural province representative of many areas of Vietnam, to determine the association of 25-OHD in late pregnancy with infant growth and developmental outcomes at 6 months of age. Our secondary objective was to determine whether maternal 25-OHD status was associated with infant birth outcomes.

## Subjects and Methods

### Study Design

This was an observational cohort study of 960 women who had previously participated in a double-blind cluster randomized controlled trial of antenatal micronutrient supplementation in rural Vietnam. Our main exposure of interest was maternal vitamin D concentration at 32 weeks gestation. Our primary outcomes of interest were infant cognitive, motor, socio-emotional and language scores using the Bayley Scales of Infant Development, 3^rd^ Edition (BSID III), and infant length-for-age z scores at 6 months of age. Secondary outcomes were birth weight and risk of preterm delivery.

### Study site and participants

The study was undertaken in Ha Nam province in northern Vietnam (latitude 20.2, climate zone- tropical) [30]. Ha Nam has a population of approximately 820,100 people, with most residents still working in subsistence agriculture. Diet consists mainly of rice, meat and vegetables. In 2010, the average annual per capita income was $USD 800 [31], [32].

Participants were all women and infants previously enrolled in a cluster randomised trial, which took place between 28^th^ September 2010 and 8^th^ Jan 2012 (Australia New Zealand Clinical Trials Registry number: 12610000944033). For the original cluster randomised trial, women were randomised to receive either (1) one tablet of iron-folic acid (IFA) taken daily (60 mg elemental iron/0.4 mg folic acid per tablet, 7 tablets per week); (2) one capsule of IFA taken twice a week (60 mg elemental iron/1.5 mg folic acid per capsule; 2 capsules per week); or (3) one capsule of multiple micronutrients (MMN) taken twice a week (60 mg elemental iron/1.5 mg folic acid per capsule/plus zinc 20 mg, iodine 300 ug, copper 4 mg, selenium 130 ug, niacin 36 mg, folic acid 1.5 mg, Vitamins A 1.6 mg, B1 2.8 mg, B2 2.8 mg, B12 5.2 ug, C 140 mg, D 400 IU, E 20 mg;2 capsules per week.) [33].

**Figure 1:**
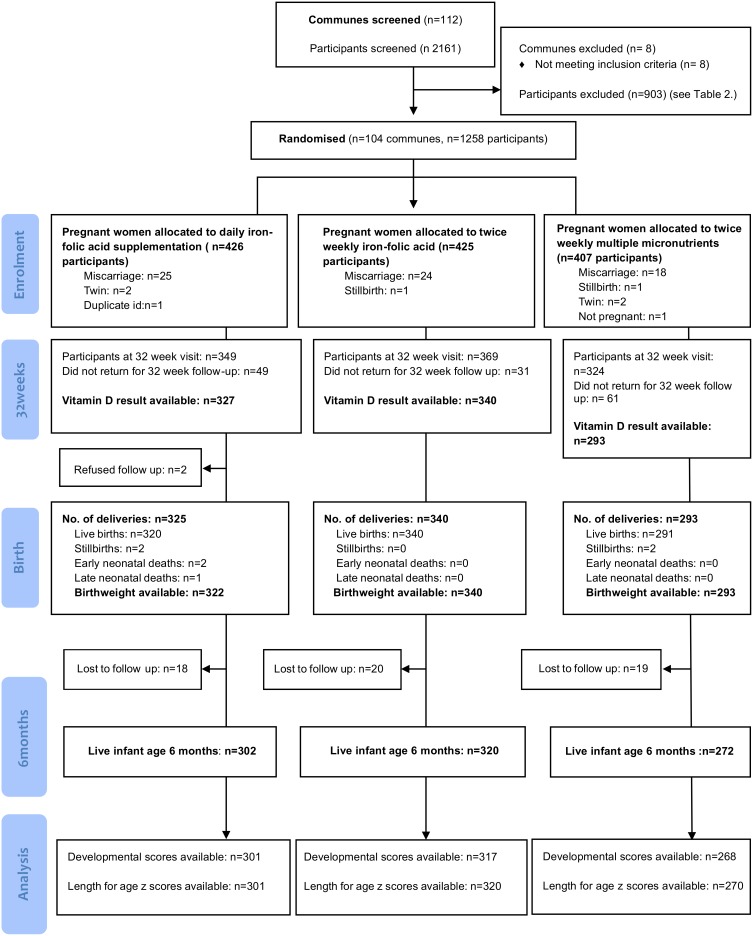
Flow diagram.

The primary objective of the original trial was to compare the effect of twice weekly antenatal provision of a) iron-folic acid supplementation or b) multiple micronutrient supplementation with daily provision of antenatal iron-folic acid supplementation, on maternal and infant outcomes. Inclusion criteria were residence in trial communes; age >16 years, confirmed pregnancy <16 weeks gestation and registration with the commune health station. Women were excluded if they had a high-risk or multi-fetal pregnancy (confirmed on palpation or ultrasound); a significant medical condition; or if their haemoglobin concentration was <80 g/L at enrolment. In total 1258 women were enrolled into the original trial. Figure 1 summarizes the recruitment, randomization and participation of subjects in the trial. The results of this trial have been previously published [34].

### Data collection and outcomes

This study was conducted between January 2011 and January 2012. Maternal serum 25-OHD was measured in women at 32 weeks gestation, and infant anthropometric and developmental measurements were performed at 6 months of age (Figure 1). Research staff who examined children at 6 months of age were blinded to maternal 25-OHD status.

### Maternal characteristics

Maternal sociodemographic factors were assessed using a standardized questionnaire administered by trained research staff at enrolment. The questionnaire included information on demographics, maternal occupation, education, marital status and pregnancy history.

### Birth outcomes

Gestational age at birth was calculated from estimated gestational age recorded by transabdominal ultrasound performed at the District Hospital if available, otherwise it was calculated according to the date of the last menstruation given at enrolment. Preterm delivery was defined as birth <37 weeks and low birth weight was defined as birth weight <2500 grams. Small for gestational age was calculated as weight below the 10th percentile for gestational age.

### Infant Developmental Assessment

Measurement of infant development was performed at 6 months of age using BSID III [35]. Bayley Scales were translated in the Vietnamese language, however no questions were changed in terms of content. Questions were translated from English into Vietnamese and back-translated to English for verification. The instrument was previously pilot tested in Hanam province and found to be comprehensible and meaningful in rural Vietnam. BSID III administrators were community-based psychologists experienced in early child development assessment, and were trained by a local Vietnamese expert in BSID III following the guidelines of the BSID 3rd Edition Manual [35]. The BSID III was used to conduct direct infant developmental assessments for cognitive, language and motor domains, and rating of mothers determined the socio-emotional and adaptive behaviour scores. Individual raw scores for cognitive, language, motor, socio-emotional and adaptive behaviour were converted into composite scores based on the guidelines of the BSID 3^rd^ Edition Manual, and used in the final analysis.

### Anthropometric measurements

Maternal height was measured with a portable stadiometer (Seca 214, Hamburg, Germany), and maternal and infant weight with a mother-infant scale (Seca 872, Hamburg, Germany). Infant length was measured with a portable Shorr board (Shorr productions, USA). Infant length for age z scores were calculated using WHO Anthro (version 3.2.2, January 2011) [36]. Research staff recorded triplicate measurements of anthropometric measures, a second observer checked all measurements, and the median measurement was used for analysis. Stunting was defined as length-for-age z scores < two standard deviations below WHO growth standards. Body mass index (BMI) was calculated as weight in kilograms divided by height in metres squared [37].

### Vitamin D Analyses

Five mls of venous blood was collected from each pregnant woman at 32 weeks gestation, and one ml of venous blood was collected from each infant at 6 months of age. Serum samples were frozen at −20°C and transported to the Alfred Hospital, Melbourne, and analysis for vitamin D was performed on those with a sufficient volume of blood. Solid Phase Extraction (SPE) using Waters Oasis uElution SPE plates was used as a pre-treatment step to release 25-OHD binding protein. Extracted patient samples were then analysed using a WatersACQUITY UPLC with an ACQUITY BEH Phenyl column (2.1×50 mm) with a water/methanol/ammonium acetate gradient. A Waters TQD mass spectrometer was used to quantify 25-OHD2 and 25-OHD3. The coefficient of variation for 25-OHD3 was 9.9% at 42 nmol/L and 9.6% at 96 nmol/L. The coefficient of variation for 25-OHD2 was 12% at 42 nmol/L and 8.8% at 94 nmol/L. Vitamin D levels were categorized as replete (≥75 nmol/L), insufficient (≥37.5 nmol/and<75 nmol/L) or deficient (<37.5 nmol/L) [12]. (1 nmol/L 25-OHD  = 0.4 ng/ml 25-OHD).

### Ethics statement

The study was approved by the Melbourne Health Human Research Ethics Committee and the Hanam Provincial Human Research Ethics Committee. The original cluster randomised trial is registered in the Australia New Zealand Clinical Trials Registry: 12610000944033. Written informed consent was collected from all participants prior to enrolment and permission was obtained from the Ministry of Health to transport biological samples across an international border.

### Statistical methods

Data were analysed using Stata, Version 12 (StataCorp, College Station, TX, USA). Categorical data are presented as percentages with frequency and continuous data are presented as mean and standard deviation (SD). Multivariable linear regression was performed to derive estimates of mean differences (95% confidence intervals (CI)) of the continuous outcome data (developmental composite scores of the infant at 6 months, gestational age and anthropometric measurements) associated with maternal vitamin D concentrations at 32 weeks, and multivariable logistic regression was used to assess the associations between maternal vitamin D concentrations and the binary outcomes (preterm delivery, low birth weight, small for gestational age and infant stunting). The association between maternal vitamin D concentrations and each of the outcome variables was assessed for linearity both visually and by comparing regression models with a categorized and pseudo-continuous form of maternal vitamin D concentrations (quintile groupings) using the likelihood ratio test. Confounders adjusted for in the multivariable regression analyses were determined a priori and included maternal age, education, gravidity, maternal body mass index (as a continuous variable), month of sampling of and micronutrient intervention. Maternal depression was also included in the analyses for infant developmental outcomes.

To account for clustering at the commune level, robust standard errors were calculated using the Huber-White Sandwich estimator. To further investigate the presence of a potential threshold effect of vitamin D concentration on infant developmental outcomes [24], secondary statistical analyses were performed on maternal vitamin D concentration categorized as deficient (<37.5 nmol/L), insufficient(≥37.5 nmol/L and <75 nmol/L) or replete (≥75 nmol/L). The level of statistical significance was 0.05.

## Results

**Figure 2:**
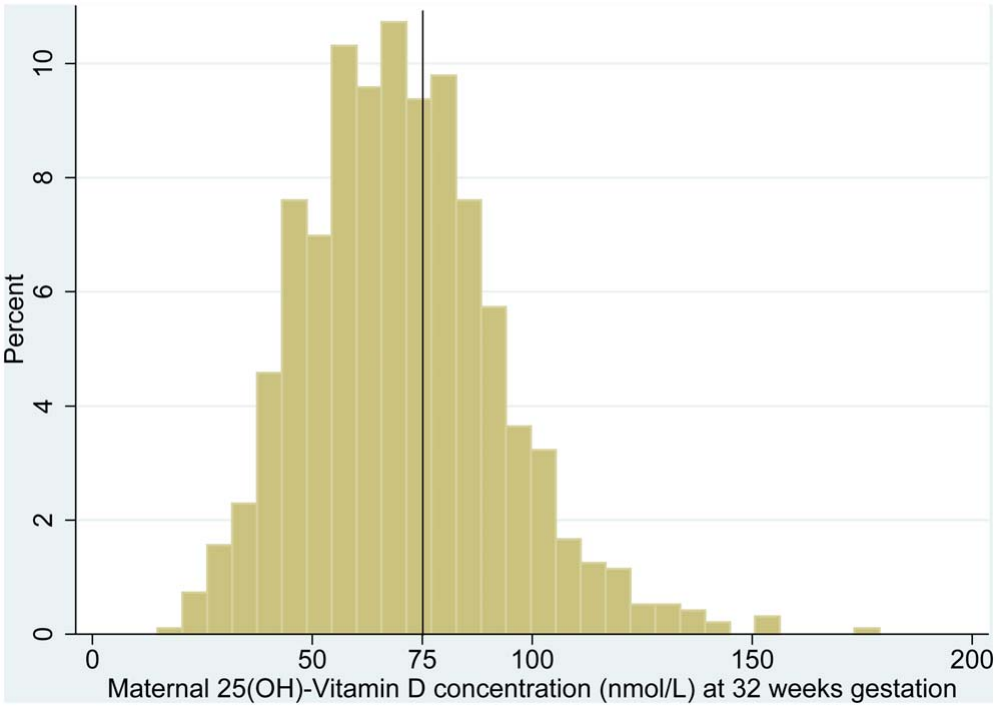
Maternal 25(OH)-Vitamin D concentration (nmol/L) at 32 weeks gestation.

The study profile is presented in Figure 1. A total of 2161 women were screened and 1258 pregnant women were enrolled into the original cluster randomised trial (Table 1). Serum 25-OHD results were available for 960 (92%) of the 1042 women who presented at the 32 week follow up visit. Baseline maternal sociodemographic and anthropometric characteristics are presented in Table 2. Sixty percent (582/960) of women had vitamin D concentrations less than 75 nmol/L in late pregnancy. Figure 2 shows the distribution of vitamin D concentration among pregnant women at 32 weeks gestation.

**Table 1:** Screening, eligibility and enrolment, of trial participants.

**Screened from Commune lists**	**2161**
Not pregnant	13
Miscarried before enrollment	42
Complicated pregnancy/sickness	12
Over eligible gestational age (>16 weeks)	628
Absent at enrollment time	51
Migrated from province after screening	31
**Eligible population**	**1384**
Excluded at enrolment	
Work commitments, could not commit to trial protocol	90
Refused	36
**Enrolled**	**1258**

**Table 2:** Distribution of baseline maternal sociodemographic, anthropometric characteristics, and maternal serum 25-OHD concentration (nmol/L) at 32 weeks gestation.

Maternal Characteristic	Number (%)	Mean maternal serum 25-OHD concentration nmol/L [SD]
Overall	960 (100)	70.5 [22.2]
Maternal age at enrolment (years)		
<20	37 (3.9)	70.2 [21.4]
20–24	298 (31)	72.3 [21.9]
25–29	400 (41.7)	69.1 [21.5]
30–34	152 (15.8)	69.5 [21.0]
≥35	73 (7.6)	72.6 [29.4]
Maternal height		
≥145 cm	930 (97.0)	70.3 [22.2]
<145 cm (short maternal stature)	29 (3.0)	75.8 [24.4]
Maternal body mass index		
Underweight (<18.5 kg/m^2^)	251 (26.2)	70.7 [22.4]
Normal (18.5–25 kg/m^2^)	655 (68.3)	69.8 [21.4]
Overweight (>25 kg/m^2^)	53 (5.5)	77.4 [29.7]
Educational level		
Primary school	146 (15.2)	73.0 [23.5]
Secondary school	643 (67.0)	70.9 [22.3]
University/college	171 (17.8)	66.4 [20.2]
Occupation		
Farmer/housewife	509 (53.0)	74.1 [22.6]
Factory worker/trader	324 (33.8)	66.6 [22.1]
Government official/clerk	127 (13.2)	65.9 [18.4]
Gravidity		
Primigravida	296 (30.8)	67.7 [21.3]
Multigravida	664 (69.2)	71.7 [22.5]
Type of supplement taken during pregnancy		
Daily IFA supplements	327 (34.1)	67.8 [21.5]
Twice weekly IFA supplements	340 (35.4)	68.0 [21.5]
MMN supplements	293 (30.5)	76.3[23.8]

IFA = Iron folic acid, MMN = Multiple micronutrients, SD = Standard Deviation.

### Maternal vitamin D and birth outcomes

Table 3 summarizes birth outcomes related to maternal vitamin D in late pregnancy. A trend towards an association with preterm delivery (Odds Ratio (OR) 0.77 per 25 nmol/L increase in vitamin D concentration, 95% CI 0.57 to 1.05) and small for gestational age (Odds Ratio (OR) 1.37 per 25 nmol/L increase in vitamin D concentration, 95% CI 0.98 to 1.91) was observed. For the 49.5% of infants with a measurement of head circumference, there was an inverse relationship between infant head circumference at birth and maternal 25-OHD (MD of −0.35 cm per 25 nmol/L increase in vitamin D concentration, 95% CI −0.62 to −0.09).

**Table 3:** Birth outcomes associated with maternal serum 25-OHD concentration (nmol/L) at 32 weeks gestation (unadjusted and adjusted models).

		Unadjusted model		Adjusted model 1		Adjusted model 2	
Birth outcomes	Mean [SD] or No.(%)	Mean difference^1^^,^ 3 or Odds Ratio^2^^,^ 3 (95% CI)	P value	Mean difference^1^^,^ 4 or Odds Ratio^2^^,^ 4 (95% CI)	P value	Mean difference^1^^,^ 5 or Odds Ratio^2^^,^ 5 (95% CI)	P value
Birth weight (g)	3168.6 [392.3]	−11.9 (−40.0 to 16.3)	0.41	−21.0^1^ (−51.2 to 9.3)	0.17	−18.9 (−49.0 to 11.3)	0.22
Birth length (cm)	49.1 [2.61]	−0.05 (−0.34 to 0.24)	0.73	−0.08^1^ (−0.44 to 0.27)	0.64	−0.07 (−0.43 to 0.29)	0.70
Head circumference (cm)	32.7 [2.12]	−0.27 (−0.49 to −0.03)	**0.03**	−0.38^1^ (−0.64 to −0.12)	**0.01**	−0.35 (−0.62 to −0.09)	**0.01**
Low birth weight (<2500 g)	27 (2.8%)	1.03 (0.67 to 1.59)	0.89	1.11^2^ (0.77 to 1.61)	0.57	1.15 (0.74 to 1.80)	0.54
Gestational age (weeks)	39.2 [1.87]	0.07 (−0.06 to 0.20)	0.31	0.08 (−0.09 to 0.25)	0.38	0.07 (−0.10 to 0.24)	0.42
Preterm delivery (<37 weeks)	103 (10.7%)	0.78 (0.61 to 1.00)	0.054	0.60^2^ (0.57 to 1.05)	0.09	0.77 (0.57 to 1.05)	0.09
Small for gestational age	50 (5.3)	1.24 (0.91 to 1.68)	0.17	1.33 (0.94 to 1.87)	0.11	1.37 (0.98 to 1.91)	0.07

1 Values are estimated mean difference for each outcome associated with a 25 nmol/L increase in vitamin D concentration (95% confidence interval).

2 Values are relative changes in the odds for each outcome associated with a 25 nmol/L increase in vitamin D concentration (95% confidence interval).

3 Unadjusted model.

4 Model adjusted for micronutrient intervention, maternal body mass index, gravidity, and clustering at commune level.

5 Model adjusted for maternal age, education, month of sampling of maternal vitamin D, micronutrient intervention, maternal body mass index, gravidity, and clustering at commune level.

SD = standard deviation; CI = Confidence Interval.

### Maternal vitamin D concentration and infant development at 6 months of age

No association between overall maternal vitamin D concentration and developmental composite scores was observed (Table 4). However, lower language composite scores were seen in infants born to women with vitamin D deficiency (<37.5 nmol/L) compared to those born to women who were vitamin D replete (≥75 nmol/L) (MD −3.48, 95% CI −5.52 to −1.44) (Table 5).

**Table 4:** Developmental composite scores at 6 months of age associated with maternal serum 25-OHD concentration (nmol/L) at 32 weeks gestation.

		Unadjusted model		Adjusted model 1		Adjusted model 2	
Domain	Mean developmental composite score [SD]	Mean difference^1^^,^ 2 (95% CI)	P value	Mean difference^1^^,^ 3 (95% CI)	P value	Mean difference 1 ^,^ 4 (95% CI)	P value
Cognitive	104.5 [10.1]	−0.47 (−1.22 to 0.27)	0.21	−0.39 (−1.19 to 0.40)	0.30	−0.43 (−1.23 to 0.37	0.29
Language	97.8 [7.6]	−0.02 (−0.58 to 0.55)	0.96	0.04 (−0.51 to 0.59)	0.89	0.08 (−0.47 to 0.63)	0.77
Motor	110.9 [13.4]	0.03 (−0.97 to 1.03)	0.95	0.25 (−0.71 to 1.21)	0.61	0.05 (−0.92 to 0.64)	0.92
Socio-emotional	79.6 [9.8]	−0.12 (−0.85 to 0.61)	0.75	−0.06 (−0.81 to 0.70)	0.88	−0.14 (−0.92 to 0.64)	0.71

1 Values are estimated mean difference for each outcome associated with a 25 nmol/l increase in vitamin.

D concentration (95% confidence interval).

2 Unadjusted model.

3 Model adjusted for micronutrient intervention, maternal education, and clustering.

4 Model adjusted for maternal age, education, month of sampling of maternal vitamin D, micronutrient.

intervention, maternal body mass index, gravidity, post-partum depression and clustering at commune level.

SD = standard deviation, CI = Confidence Interval.

**Table 5:** Developmental composite scores in infants at 6 months of age according to maternal serum 25-OHD concentration levels at 32 weeks gestation.

	Unadjusted model					Adjusted model			
Domain	Vitamin D replete (≥75 nmol/L)	Vitamin D insufficient (≥37.5, <75 nmol/L)^1^^,^ 2	P value	Vitamin D deficient (<37.5 nmol/L)^1^^,^ 2	P value	Vitamin D insufficient (≥37.5, <75 nmol/L)^1^^,^ 3	P value	Vitamin D deficient (<37.5 nmol/L)^1^^,^ 3	P value
Cognitive	Reference	0.86 (−1.07 to 2.79)	0.38	−0.22 (−3.35 to 2.92)	0.89	0.58 (−1.24 to 2.40)	0.53	−0.44 (−3.21 to 2.33)	0.75
Language	Reference	1.14 (−0.31 to 2.58)	0.12	−3.14 (−5.49 to −0.79)	**0.01**	0.98 (−0.47 to 2.42)	0.18	−3.48 (−5.52 to −1.44)	**0.01**
Motor	Reference	0.11 (−2.46 to 2.69)	0.93	−0.49 (−4.68 to 3.71)	0.82	−0.27 (−2.76 to 2.23)	0.83	−0.57 (−5.21 to 4.07)	0.81
Socio-emotional	Reference	0.35 (−1.53 to 2.24)	0.71	−0.56 (−3.63 to 2.51)	0.72	0.14 (−0.1.56 to 1.85)	0.89	−0.45 (−3.7 to 2.8)	0.79

1 Values are estimated mean difference (95% confidence interval).

2 Unadjusted model.

3 Model adjusted for maternal age, education, month of sampling of maternal vitamin D, micronutrient intervention, maternal body mass index, gravidity, maternal depression and clustering.

### Maternal vitamin D and anthropometric measurements at six months of age

An inverse association between maternal 25-OHD and infant length-for-age z scores at 6 months of age was demonstrated (MD −0.09 per 25 nmol/L increase in vitamin D concentration, 95% CI −0.12 to −0.02) (Table 6).

**Table 6:** Infant anthropometric outcomes at 6 months of age associated with maternal serum 25-OHD concentration (nmol/L) at 32 weeks gestation.

		Unadjusted model		Adjusted model	
Infant anthropometric outcomes	Mean [SD] ornumber(%)	Mean difference^1^^,^ 3 or Odds Ratio^2^^,^ 3 (95% CI)	P value	Mean difference^1^^,^ 4 or Odds Ratio^2^^,^ 4 (95% CI)	P value
Length (cm)	66.0 [2.28]	−0.10 (−0.27 to 0.06)^1^	0.23	−0.11 (−0.26 to 0.05) 1	0.19
Weight (kg)	7.71 [0.98]	−0.02 (−0.09 to 0.05) 1	0.61	−0.03 (−0.10 to 0.04) 1	0.45
Head circumference (cm)	42.6 [1.43]	0.003 (−0.10 to 0.11) 1	0.95	−0.01 (−0.10 to 0.11) 1	0.95
HAZ	−0.57 [0.91]	−0.09 (−0.15 to −0.02)^1^	**0.02**	−0.09 (−0.12 to −0.02)^1^	**0.02**
Stunted	55/891 (6.2)	0.92 (0.68 to 1.26) 2	0.62	0.89 (0.59 to 1.32) 2	0.52

1 Values are estimated mean difference for each outcome associated with a 25 nmol/L increase in vitamin D.

concentration (95% confidence interval).

2 Values are relative changes in the odds for each outcome associated with a 25 nmol/L increase in vitamin D concentration (95% confidence interval).

3 Unadjusted model.

4 Model adjusted for maternal age, education, month of sampling of maternal vitamin D, micronutrient intervention, maternal body mass index, gravidity, and clustering.

## Discussion

These results indicate that there is a high prevalence of vitamin D insufficiency in pregnant women residing in rural Ha Nam Province, Vietnam. Our findings show that low vitamin D levels in late pregnancy (<37.5 nmol/L) are associated with reduced infant language development at six months of age.

An association between low maternal vitamin D concentration and language impairment has previously been suggested by Whitehouse et al. in a study involving a cohort of pregnant women and school-aged children in Australia. They reported that the risk of having a child with language difficulties at 5 and 10 years of age increased by two-fold in women with an antenatal vitamin D concentration ≤46 nmol/L [24]. In this study, vitamin D concentration was measured early, at 18 weeks gestation, and receptive language was assessed using the Peabody Picture Vocabulary Test. Gale et al., measured maternal vitamin D concentration during the third trimester and assessed infants using the Weschler Abbreviated Scale of Intelligence (n = 178), and found no association between maternal vitamin D levels and full scale, verbal or performance IQ at 9 years of age [38].

In our previous publication [34], no significant differences in infant language or developmental outcomes were observed in infants born to women who received twice weekly multiple micronutrients compared to those who received daily iron folic acid. Although vitamin D concentration was significantly higher in the twice weekly multiple micronutrient group compared to daily iron folic acid group, (mean [SD] of 76.3 [23.8] versus 67.8 [20.6] nmol/L), the prevalence of vitamin D deficiency (<37.5 nmol/L) was similar (4.9% (16/326) versus 3.1% (9/293). Our finding of an association between maternal vitamin D and infant language composite scores, that was only observed when maternal vitamin D levels fell below 37.5 nmol/L in late pregnancy, raises the possibility that there is a threshold effect, and that language impairment is a feature of more marked vitamin D deficiency. The observed association between antenatal vitamin D concentration and infant development is plausible as vitamin D has an important role in neurodevelopment through cell differentiation, cytokine regulation, neurotransmitter synthesis and anti-oxidant activity, and it is has been suggested that *in utero* exposure to vitamin D deficiency during the second and third trimesters may affect the development of the Perisylvian structures, which are responsible for language in children [24], [39].

There has also been interest in the link between autism and vitamin D deficiency. Recent research suggests that vitamin D deficiency during pregnancy or early childhood may be an environmental trigger for autism spectrum disorder in infants genetically predisposed to autism, either through its effect on neuronal function and brain development, or through gene interaction and regulation. However larger, high quality trials are required [10], [25], [26].

We observed an inverse relationship between length-for-age z scores at 6 months of age and overall maternal vitamin D concentration, although the estimated magnitude of change associated with an increase in vitamin D of 25 nmol/L was small. Several studies have found an association between maternal vitamin D status and post-natal growth, but results are inconsistent and the majority are observational studies that have been carried out in developed countries. Previous authors have suggested that accelerated growth in length may occur during an infant’s first year of life in infants born to mothers with vitamin D <30 nmol/L, as infant vitamin D levels increase postnatally, through post-natal vitamin D supplementation [5]. Other studies have shown no differences in weight or height across quartiles of vitamin D status during infancy [40].

We found increasing maternal vitamin D concentration showed a trend towards a reduced risk of preterm delivery. A positive association between maternal vitamin D concentration and gestational duration has previously been described [41], [42], and Morley et al. [43] demonstrated a significantly shorter gestation (0.7 weeks) among women with 25-OHD <28 nmol/L living in Australia, compared to those with higher levels. A pooled analyses of two previous studies [17] showed no significant link between vitamin D supplementation and preterm delivery, however the two studies had small sample sizes (350 and 180 participants) [4], [44], with relatively large loss to follow up (30%). One potential explanation for the association with preterm birth, is an increased risk of vaginal infection with low antenatal Vitamin D concentration, as bacterial vaginosis is a known risk factor for preterm birth. The association between vitamin D concentration and vaginal infection has previously been described by several authors [45], [46], [47].

We found no association between vitamin D deficiency and infant birth weight, low birth weight and birth length. This is in keeping with a Cochrane review of data from three trials showing that vitamin D supplementation during pregnancy did not affect birth weight or birth length [13]. An inverse association between birth head circumference and maternal vitamin D was observed, however this finding did not persist at 6 months of age. This result needs to be interpreted with caution, as head circumference at birth was recorded in less than half the infants. These findings are in contrast to Aghajafari et al. [12] who assessed the effect of 25-OHD levels on pregnancy outcomes and birth variables in a systematic review of four studies, and found no significant difference in head circumference in infants born to women with 25-OHD concentrations less than 37.5 nmol/L during pregnancy. Our findings need to be interpreted with caution, as head circumference at birth was recorded in less than half the infants. Further exploration is required.

There are no standardised definitions for vitamin D thresholds in pregnancy (deficiency, insufficiency and sufficiency), however a recent meta-analysis defined insufficiency for pregnancy outcomes as less than 75 vnmol/L, and insufficiency for birth variables as less than 37.5 nmol/L [12]. The reported prevalence and definitions of vitamin D insufficiency/deficiency in Vietnamese women has varied in previous studies, ranging from 7% to 46% [27], [28], [29]. In our trial, even though the majority of women worked as farmers, we observed a very high prevalence (60%) of women with vitamin D levels less than 75 nmol/L. This is likely to be the result of cultural influences, with the common use of face masks and long sleeved gloves, and the perception that white skin is ‘attractive’, leading to restricted exposure to sunlight [28]. Some authors have also suggested that the cut off for sufficient vitamin D levels during pregnancy should be as high as 100–150 nmo/L [10], [11], and therefore the problem of vitamin D deficiency in Vietnam and other South East Asian countries may be even more widespread than previously thought.

To our knowledge, our study is the largest and most comprehensive study on maternal vitamin D status to be conducted in Vietnam. Our study was conducted in a rapidly developing rural area, which is representative of many other areas of Vietnam. The large sample size and rigorous trial design of the original cluster randomised controlled trial also allowed us to follow the cohort of infants past the neonatal period into infancy, to document important growth and developmental outcomes within the first 6 months of life.

A limitation of our study was that we investigated several outcome measures and therefore the evidence against the null hypothesis observed for language development may be due to Type I error. However, similar findings in previous studies [24], [38], [41], [42], [43] and the increasing evidence for vitamin D deficiency as a potential environmental risk factor for autism [10], [25], [26] suggest that these associations should not be discounted. The differences we observed were also of magnitudes of clinical importance; thus further exploration in larger cohorts, preferably in non-western settings, is indicated.

## Conclusions

Our study indicates that low levels of vitamin D during pregnancy are of concern in rural Vietnam, and are associated with impaired language development at 6 months of age. Our results strengthen the evidence for giving vitamin D supplementation during pregnancy and highlight the need for further research to explore the impact of supplementation on longer term child growth and developmental outcomes, particularly with regard to language.
